# The Impact of the Third Wave of the COVID-19 Pandemic on the Elderly and Very Elderly Population in a Tertiary Care Hospital in Portugal

**DOI:** 10.7759/cureus.22653

**Published:** 2022-02-27

**Authors:** Maria João Palavras, Cátia Faria, Patrícia Fernandes, Alexandra Lagarto, Ana Ponciano, Filipa Alçada, Maria Jesus Banza

**Affiliations:** 1 Internal Medicine, Centro Hospitalar de Leiria, Leiria, PRT; 2 Physical Medicine and Rehabilitation, Centro Hospitalar de Leiria, Leiria, PRT

**Keywords:** very elderly, treatment, portugal, mortality, elderly, covid-19, comorbidities

## Abstract

Background: COVID-19 poses a significantly more serious threat to adults aged 65 and above, with a higher mortality rate. This study aims to describe the outcome of COVID-19 patients in the elderly and very elderly population admitted to a tertiary care Portuguese hospital. The authors defined the elderly population (65 to 79 years) and the very elderly population (≥ 80 years).

Methods: We conducted a retrospective observational single center study in the internal medicine ward of a tertiary hospital from November 1, 2020 to January 31, 2021. All COVID-19 patients aged over 65 years were enrolled.

Results: Of the 824 patients with SARS-CoV-2 infection, 586 (71%) were aged above 65 years. Of them, 61.7% were very elderly and 32.9% were elderly. The hospital recorded 53 (27.5%) deaths in the elderly group and 182 (46.3%) in the over-80 group. In the elderly population, only 32 patients had critical illness compared to the 79 in the very elderly group. In addition to respiratory complications, acute kidney failure and liver dysfunction were noted. In both groups, mortality was higher when there was acute kidney injury (AKI). With respect to treatment, dexamethasone and azithromycin did not show a statistically significant difference between the groups. The need for oxygen therapy over 4L/min, high-flow therapy, and mechanical invasive ventilation was related to higher mortality in both groups.

Conclusion: The very elderly group had a higher number of deaths compared to the elderly group due to multiple comorbidities. Respiratory failure was the most frequently occurring complication. Surprisingly, dexamethasone and azithromycin therapy did not show a statistically significant effect in both age groups despite their current widespread usage in COVID-19 treatment worldwide.

## Introduction

Severe acute respiratory syndrome coronavirus 2 (SARS-CoV-2), has created a matchless health crisis in this century. The level of suffering experienced by humankind in the last two years can perhaps only be matched by the one inflicted by the Spanish flu pandemic. Even with the total number of deaths amounting to 1,813,188 by December 31, 2020 (data reported by the World Health Organization), the number of deaths may possibly be underestimated due to the undercount of deaths indirectly caused by COVID-19 as well as unreported deaths in countries with less developed health systems [1-3].

COVID-19 classically presents with general symptoms that are associated with viremias, such as myalgia, headache, fever, general exhaustion, as well as respiratory symptoms, namely dyspnea, cough, and chest pain. However, some other clinical findings may be seen as well, notably extrapulmonary, such as diarrhea, nausea, hyposmia, hypogeusia, liver dysfunction, and frequently in the elderly population, altered mental status [2-4].

Although COVID-19 poses a risk to people of all ages, it is a more serious threat to adults aged 65 and above, who are disproportionately impacted and account for the majority of patients with severe disease, prolonged hospitalization, admission to ICUs, and death. In the USA, more than 80% of deaths occurred in patients older than 65 years [2]. In Italy, one of the countries that were most affected in the first months of the pandemic, 25.3% of the infected were over 65 years old and 55.3% of the deaths involved people aged over 80 [4]. Current evidence has that old age, male sex, and conditions such as obesity, hypertension, and other cardiovascular diseases predict a poor prognosis and higher risk of death [5].

Since subpopulations of the elderly (65 to 79 years) and very elderly (≥ 80 years) are at a higher risk, it is crucial to analyze what lies behind this frailty. Therefore, in this study, we aim to describe the experience of the elderly and very elderly COVID-19 population in a tertiary hospital related to risk factors associated with negative outcomes and prognosis.

## Materials and methods

A retrospective observational single-center analysis was performed in a tertiary care hospital in Portugal. Data were collected from the clinical records of all COVID-19 patients admitted to the internal medicine department, between November 1, 2020 and January 31, 2021, corresponding to the third wave. After reviewing clinical notes, surgical patients (patients who were admitted solely for a surgical procedure) were excluded. Only those aged 65 years above were analyzed. Those who were initially admitted to critical care units were also excluded.

Disease severity was defined according to WHO guidelines. The study protocol follows the Declaration of Helsinki. All the patients were evaluated taking into account the clinical files from hospital admission. Demographic data, diagnosis at admission, comorbidities, as well as clinical and laboratorial data, including renal and respiratory failure were included. Discharge status was also collected data. 

The primary outcome was the quantified death rate and the risk factors associated with higher mortality, in our population infected by SARS-CoV-2. Complications of SARS-CoV-2 infection associated with higher mortality were determined in both samples were also evaluated, and the association of treatment with survival in both samples was also analyzed. Group comparisons between the elderly and very elderly were performed using the Student’s t-test to compare continuous with categorical variables. The chi-square test for categorical variables and Pearson’s correlation was used to compare two continuous variables. A p-value of less than 0.05 was considered significant. Statistical analysis was performed using SPSS software version 27.0 (IBM, Armonk, NY, USA).

## Results

A total of 824 cases of SARS-CoV-2 infection were registered in this period. Of them, 238 patients did not meet the inclusion criteria. Out of the remaining, 586 (71%) were ≥ 65 years old and 393 (29%) were above 80 years old. Patients aged ≥ 80 years had a mean age of 88 (87.5 ± 5.0) years and those aged between 65 and 79 had a mean age of 74 (73.6 ± 4.0) years. Of the individuals aged above 80 years, 43% (n = 169) were male. And of those between 65 and 79 years, 49.7% were male (n = 96).

Of those aged between 65 and 79 years, 29% (n = 56) had no significant disability (defined as having a Modified Rankin Score of 0). However, in the very elderly population, a larger proportion of patients 134 (34.2%) had severe disabilities that required constant nursing care and attention (mRankin 5) (Table 1).

**Table 1:** Demographic, epidemiological, and clinical characteristics of 586 COVID-19 hospitalized adults ≥65 years old GRF: Glomerular Filtration rate. HIV: human immunodeficiency virus. SD: standard deviation *2021 ESC Guidelines for the diagnosis and treatment of acute and chronic heart failure.  **European AIDS Clinical Society Guidelines Version 11.0, October 2021.

Demographic	65 to 79 years old, n (%)	≥80 years old, n (%)
Total	193 (33%)	393 (67%)
Female	97 (50.30%)	224 (57%)
Male	96 (49.70%)	169 (43%)
Median age (years ±(SD))	73.6 ±(4.02) years	87.50 (±5.03) years
Modified Rankin Score		
0	46 (23.8 %)	22 (5.6%)
1	56 (29.0%)	53 (13.5%)
2	11 (5.7%)	34 (8.6%)
3	26 (13.5%)	56 (14.2%)
4	24 (12.4%)	94 (24%)
5	30 (15.5%)	134 (34.1%)
Deaths	53 (28%)	182 (46.3%)
Comorbidities		
Chronic kidney disease (GRF<60mL/min/1.73m^2^)	19 (9.8%)	93 (23.6%)
Chronic liver disease*	6 (3.1%)	3 (0.8%)
Diabetes mellitus (HbA1>6.5%)	76 (39.4 %)	119 (30%)
Heart failure*	30 (15.5%)	127(32.6%)
HIV**	2 (1.0%)	1 (0.3%)
Hypertension (TA > 140/90mmHg)	139 (72%)	304 (77.4%)
Immunosuppressant drugs (steroids, anti-TNF alpha, biological, chemotherapy)	4 (2.1%)	8 (2%)
Lung Disease (asthma, COPD, pulmonary fibrosis, lung cancer)	44 (22.8%)	79 (20.1%)
Neoplasm	24 (12.4%)	47 (12%)
Obesity (IMC>25kg/m^2^)	43 (22.3%)	70 (17.8%)
Smoker	13 (6.8%)	7 (1.8%)

The main comorbidities observed in elderly patients were hypertension (72%), diabetes mellitus (39.4%), and lung disease (22.8%). In the very elderly population, the main comorbidities were more frequent hypertension (77.4%) heart failure (32.6%), and diabetes mellitus (30%).

The hospital recorded 53 (28%) deaths in the elderly group and 182 (46.3%) in the above 80 years (Table 1). Patients with mild illness were admitted due to lack of isolation conditions at home and did not need oxygen supplements. In the elderly population, only 32 patients (16.3%) had a critical illness from COVID-19. Similarly, 79 (20%) very elderly people met critical illness criteria (Table 2).

**Table 2:** Clinical syndrome in 586 COVID-19 hospitalized >65 years

Clinical syndrome (WHO)	65 to 79 years old, n (%)	≥80 years old, n (%)
Mild illness	44 (23%)	86 (21.9%)
Moderate	52 (27%)	91 (23.2%)
Severe	65 (33.7%)	137 (34.9%)
Critical	32 (16.3%)	79 (20%)

Moreover, 142 patients (73.6%), aged 65-79 years, with type 1 respiratory failure were registered. In the other sample, containing patients older than 80 years, 289 (73.5%) patients with type 1 respiratory failure were recorded. Death and type of respiratory failure had no relationship in the elderly population; however, in the very elderly group, mortality was higher when there was type 2 respiratory failure - chi-square: X2(2) = 14,381; p < 0.001.

Other complications were detected, such as acute kidney failure, venous and arterial thromboembolism, and liver dysfunction. Venous thromboembolism did not occur in patients in the elderly group; only one case was observed in the very elderly. Similarly, only two cases, of arterial embolism were diagnosed in the very elderly group, with 50% mortality. In both age groups, there was a higher mortality associated with the presence of acute kidney injury (AKI) - chi-square, X2(1) = 19,201; p < 0.001 for the very elderly population 80 and X2(1) = 9,915; p = 0.002 for the elderly population. Death and liver dysfunction were also associated with a poor prognosis for the elderly population, it was unrelated; for the very elderly population, mortality is higher when there was a hepatocellular injury - chi-square. X2(3) = 10,853; p = 0.005 (Table 3).

**Table 3:** Complications in COVID-19 patients hospitalized >65 years * Chi-square test was used to calculate statistical significance between the mortality and complications

Complications	65 to 79 years old, n (%)	P-value (95% CI)*	≥80 years old, n (%)	P-value (95% CI)*
Type 1 respiratory failure (pO2<60mmHg)	142 (73.6%)	0.379	289 (73.5%)	0.2
Type 2 respiratory failure (pO2<60mmHg, pCO2>45mmHg)	14 (7.3%)	0.372	32 (8.1%)	<0.001
Acute kidney failure	68 (35.2%)	0.002	194 (49.4%)	<0.001
Venous thromboembolism	0 (0%)	0	1 (0.3%)	0.283
Arterial embolism	2 (1%)	0.476	0 (0%)	0
Liver dysfunction	26 (13.5%)	0.695	59 (15.1%)	0.005

The guidelines for the treatment of SARS-CoV-2 infection at the level I care were consistent: supplemental oxygen, corticosteroids, and antibiotics, in addition to venous thromboembolism prophylaxis.

With respect to treatment, dexamethasone and azithromycin therapy did not show a statistically significant effect in both age groups. Oxygen therapy was required in 154 (79.8%) elderly people and 310 (79.3%) very elderly people. Of the 154, only six (3.1%) required invasive mechanical ventilation (IMV) (Table 4). In both age groups, there was higher mortality when oxygen therapy was required at more 4L/min and when IMV was required. Moreover, with high flow, at ≥ 80 years - chi-square. X2 (5) = 60,548; p < 0.001 for ≥ 80 and X2(5) = 52.923; p < 0.001 from 65 to 80 years. The need to use supplemental oxygen was associated with a worse prognosis.

**Table 4:** Therapies administered in 586 COVID-19 hospitalized * Chi-square test was used to calculate statistical significance between the mortality and complications

Treatment	65 to 79 years old, n (%)	pPvalue (95% CI)*	≥80 years old, n (%)	P-value (95% CI)*
Immunomodulatory therapy				
Dexamethasone iv 6mg/d	65 (33.7%)	0.865	88 (22.4%)	0.396
Antibiotics				
Azithromycin iv 500mg/d	113 (58.5%)	0.195	205 (52.3%)	0.479
Support therapy	154 (79.8%)	0.001	310 (79.3%)	0.001
Oxygen <4L/min	72 (37.3%)		141 (36.1%)	
Oxygen ≥4L/min	50 (25.9%)		123 (31.5%)	
High flow	5 (2.6%)		14 (3.6%)	
Non-invasive mechanical ventilation	21 (10.9%)		31 (7.9%)	
Invasive mechanical ventilation	6 (3.1% )		1 (0.3%)	

## Discussion

The third wave of the COVID-19 pandemic was considered the deadliest, especially for the very elderly population [6]. Most of the elderly population in Portugal is still autonomous and managed to isolate themselves in their homes. However, in the context of the very elderly population, in addition to comorbidities that make them more fragile, isolation in nursing homes that are far from their families makes recovery difficult [6,7]. The authors wanted to present a picture of the hospitalization scenario, for medical pathology, during the third wave of COVID-19. Only patients aged 65 years and above were selected.

To our knowledge, this is the first Portuguese study directly that addresses COVID mortality in the very elderly population. Very elderly adults are the main victims of COVID-19 due to their medical comorbidities and immunosenescence. As the authors were able to prove, the very elderly population had a higher number of deaths (46.3%) compared to the elderly one (28%), which confirmed the general population’s susceptibility to SARS-CoV-2 infection and is in concordance with the natural demographic data [8].

The very elderly patients also had an increased mortality rate, to the frequent occurrence of multiple comorbidities [8]. All age groups are at risk of infection from SARS-CoV-2 but very elderly people are at a significantly higher risk of mortality and severe disease following infection, with patients from this population dying at five times the average rate [8,9]. An estimated 75% of people who are above 80 years old have at least one underlying condition, placing them at increased risk of a worse prognosis of COVID-19 [8].

Elderly people appear to be more susceptible, especially to more severe forms of the disease, which increases the probability of ICU admission [9,10]. However, the low physiological reserve medical comorbidities a longer post-intensive care recovery associated with complications from IMV [11,12].

The main complications reported in patients with SARS-CoV-2 may include coagulopathy (disseminated intravascular coagulation, venous thromboembolism, elevated D-dimer, and prolonged prothrombin time); acute respiratory distress syndrome (ARDS); necrotizing pneumonia due to superinfection; acute liver, cardiac, and renal injury; septic shock; and rhabdomyolysis, but the lungs are the most affected [8,10-13]. 

In this study, the authors identified respiratory failure as the most frequently occurring complication, as COVID-19 is a pathology that primarily affects lung tissue. It was concluded that respiratory failure and death, had no relationship, in the elderly group but in the very elderly group, mortality is higher [11-13]. This is essential to immunosenescence and respiratory history (chronic obstructive pulmonary disease, asthma) that weaken the lung parenchyma [12].

Infection by SARS-CoV-2 may predispose patients to thrombotic disease; however, the pathophysiological mechanisms are not yet known. The dilemma lies in whether: hemostatic changes are a specific effect of SARS-CoV-2 or a consequence of the cytokine storm that precipitates the onset of (systemic inflammatory response syndrome) as observed in other the case of viral diseases [14,15].

In this study, venous thromboembolism was not observed in the elderly population and only one case was reported in the very elderly population. Similarly, there were only two cases of arterial embolism (in the very elderly population); one died and one survived.

Venous or arterial thromboembolismcan be explained by excessive inflammation, platelet activation, endothelial dysfunction, and stasis. As there are already several studies on thromboembolic phenomena in the context of COVID-19, physicians are already alert and introducing thromboprophylaxis [14-16]. However, the high incidence of thromboembolic complications in COVID-19 patients is a big source of concern, especially in patients who have already been administered thromboprophylaxis agents [14,15].

AKI is common among COVID-19 patients and is associated with a fatal prognosis [13]. This kidney damage is also justified by the release of inflammation mediators that reach the kidney tissue, thus compromising kidney function. Kidney damage has a worse prognosis in patients who already have chronic kidney disease. With the decrease of urinary output and ARDS, the cardiovascular volume is compromised, leading to lactic acidemia, thus aggravating respiratory acidosis despite hyperventilation [14,15].

In this study, the occurrence of AKI was strongly associated with increased mortality in both age groups. Patients with COVID-19 commonly exhibit elevated markers associated with liver injuries: AST, ALT, alkaline phosphatase, and gamma-glutamyltransferase [17]. The prognostic value of elevated liver injury markers in patients with COVID-19 remains controversial [18]. One possible mechanism underlying the liver injury observed in patients with COVID-19 is direct hepatic infection by SARS-CoV-2, and the liver is also affected by the hypoxia and cytokine storm as the lungs and kidneys in respiratory and renal failure [16,18].

In this review, oxygen therapy was required for more than 75% in both groups, and only six (3.1%) IMV. During this study, in both groups, death and ventilatory support are correlated. We propose that this happens because the need for increased use of O2 is correlated with the storm of cytokines composed of pro-inflammatory cytokines that act on cardiovascular cells, in secondary hemophagocytic lymphohistiocytosis as well as the consequent ARDS [19,20].

Several studies explained that the antiviral and anti-inflammatory properties of azithromycin are suited for patients with early-stage COVID-19 [21,22]. In vitro, azithromycin has broad antiviral activity against human viruses, including SARS-CoV and SARS-CoV-2, as it has been shown to reduce viral replication [21]. Despite these theoretical considerations, in clinical trials of azithromycin having an immunomodulatory effect, no clinical efficacy has been seen in reducing mortality, need for IMV, duration of hospital admission, or clinical status on usual outcome scores in patients admitted to hospital with COVID-19 [20-22]. However, these studies did not include very elderly people who had several comorbidities, who possibly resorted to health services late, or who probably died due to an inflammatory phenomenon.

This study has some limitations, as very elderly people already have some pathologies that make them more fragile, and during the third wave, there were not many treatment protocols or comparative studies.

## Conclusions

As this article has already reported, the infection caused by this virus is composed of pro-inflammatory cytokines that explain all the signs and symptoms of the disease, and glucocorticoids may modulate inflammation-mediated lung injury and thereby reduce progression to respiratory failure and death. Many studies have proved that the use of dexamethasone reduces the number of days of hospitalization as well as mortality in mechanically ventilated patients. These results also show that, among the patients who were receiving oxygen, the use of dexamethasone was associated with a lower risk of admission to the ICU.

However, dexamethasone and azithromycin therapy did not show a statistically significant effect in both age groups. After the first, second, and third waves have passed, the virus not only lost virulence but also its pathophysiological mechanisms are increasingly known. The authors recognize that studies in very elderly patients have many comorbidities and the low physiological reserve may lead to higher mortality. However, this is the reality of our hospital: elderly and very elderly population. Until the completion of this study, not all patients had the first dose of the vaccine against SARS-CoV-2, which may lead to lower mortality.
